# Hydra: a scalable proteomic search engine which utilizes the Hadoop distributed computing framework

**DOI:** 10.1186/1471-2105-13-324

**Published:** 2012-12-05

**Authors:** Steven Lewis, Attila Csordas, Sarah Killcoyne, Henning Hermjakob, Michael R Hoopmann, Robert L Moritz, Eric W Deutsch, John Boyle

**Affiliations:** 1 Institute for Systems Biology, Seattle, WA, USA; 2 PRIDE Group Proteomics Services Team EMBL European Bioinformatics Institute, Wellcome Trust Genome Campus, Hinxton, Cambridgeshire, UK; 3 Luxembourg Centre for Systems Biomedicine, University of Luxembourg, Luxembourg, Germany

## Abstract

**Background:**

For shotgun mass spectrometry based proteomics the most computationally expensive step is in matching the spectra against an increasingly large database of sequences and their post-translational modifications with known masses. Each mass spectrometer can generate data at an astonishingly high rate, and the scope of what is searched for is continually increasing. Therefore solutions for improving our ability to perform these searches are needed.

**Results:**

We present a sequence database search engine that is specifically designed to run efficiently on the Hadoop MapReduce distributed computing framework. The search engine implements the K-score algorithm, generating comparable output for the same input files as the original implementation. The scalability of the system is shown, and the architecture required for the development of such distributed processing is discussed.

**Conclusion:**

The software is scalable in its ability to handle a large peptide database, numerous modifications and large numbers of spectra. Performance scales with the number of processors in the cluster, allowing throughput to expand with the available resources.

## Background

The identification of peptide sequences from spectra is a computationally expensive and rate limiting task. This expense is due to the fact that the identification process typically involves each measured spectrum being matched against the likely (artificially generated) spectra for each possible peptide ion within a range of mass-to charge ratios. The problem with identification of peptides is confounded as the number of peptides is huge, and the search space increases geometrically with the number of amino acid modifications considered. This paper introduces a highly scalable strategy for overcoming these limitations, which has been purposefully built to take full advantage of a highly distributed computation framework.

The shotgun proteomics workflow has become the most widely used technique for identifying and quantifying proteins present in a biological sample in a high-throughput manner. Although many variations exist, the basic approach begins with extracting proteins from sample and digesting the proteins into peptides with a proteolytic enzyme such as trypsin. The peptides are then separated using liquid chromatography and analyzed by mass spectrometry (MS). The mass-to-charge (*m*/*z*) ratios of the peptide precursor ions are measured and the precursors are fragmented into a series of ions and measured in tandem mass spectrometry (MS/MS) mode. The resulting MS/MS spectra of these peptide ions together with the precursor *m*/*z* are searched against a database of possible peptides to determine the best match [1].

Many strategies have been proposed to help with such search techniques. Raw, embarrassingly parallel approaches are the most widely used (e.g. for X!Tandem [2], SEQUEST [3], OMSSA [4]). To increase the speed and scalability of the searching advances in high performance computing hardware have also been used to offload the searching from general to dedicated hardware (including GPUs [5] and FGAs [6]), and improvements to search algorithms have been implemented [7,8]. Some algorithms have been ported to parallel computing architectures such as MPI ( [9]). Previous parallel implementations generally scale with the number of spectra [10], however the scalability issues relating to the size of the database being searched are not addressed.

In this paper we introduce Hydra, which is designed to scale both in terms of the number of spectra and the size of the search database. Hydra makes use of Hadoop [11], which is a common and well supported framework for handling distributed computation. The Hadoop framework handles the management of a cluster of generic machines. It handles the details of taking a set of two tasks: map and reduce [12] (see methods for details) and creating the instances required to handle a given data set. Tasks are sent by the framework to specific machines and output is collected by the framework to the next task. The framework handles details such as failure and retry, generation of a distributed data system and storage and access to temporary results.

Using Hadoop for mass spectrometry based searching has been suggested before [13], where an existing MPI search engine (X!!Tandem [2]) was wrapped to work with Hadoop. However, such MPI based code does not use critical features of the MapReduce algorithm (e.g. the ability to sort data in ways that guarantee that searches are performed optimally). The algorithm introduced in this paper is a complete bottom-up rewrite of the X!Tandem code, and has been designed to take full advantage of map-reduce. Within Hydra, we implement the K-score [14], which is one of the pluggable scoring algorithms packaged with the Trans-Proteomic Pipeline [15,16] distribution of X!Tandem. The K-score is an implementation of the COMET scoring function, which produces a dot product of matched peak intensities after spectrum normalization [14]. The K-score normalization and scoring routines account for noise and unmatched peaks within the input spectra.

The advantages of the approach introduced in this paper are: ***scalability***, as Hadoop has been shown to be able to handle massive data sets in the petabyte range, and has been used to undertake complex search and analysis operations on datasets of this size. This means, as is shown in this paper, that such a framework is highly suited for the growing data sets that are becomingly searched against when undertaking MS/MS analyses; ***flexibility***, as the system can be used on a variety of commodity hardware configurations, including running across heterogeneous machines where the performance scales with additional hardware, and can be used within cloud environments (e.g. Amazon EC2) that support Hadoop; and ***reliability***, as Hadoop provides the necessary libraries to handle the critical issues of task distribution, monitoring and failure recovery (as the numbers of tasks, nodes and data sets increases, so too does the probability of task failures, which is an important issue with high throughput experiments).

In this paper we present the design for decomposition of mass spectrometry search into a collection of map/reduce tasks, and show an implementation of our proteomic search engine Hydra. Hydra is specifically designed from the ground up to work within the MapReduce algorithm. By comparison to X!Tandem we show where and how the system scales, and discuss the advantages of our approach. The software is made publicly available under an open source license.

## Implementation

The strategy advocated in this paper for ensuring scalable searches of mass spectrometry data is one that uses distributed parallel processing. This type of solution has become popular over the last five years as it allows for scalability using commodity hardware, and so both reduces the hardware and software development costs traditionally associated with high performance computing. This style of architecture allows for the development of parallel solutions across clusters of heterogeneous hardware.

With highly distributed frameworks, the times required for execution scale inversely with the number of processors allocated and directly with the size of the data set. These frameworks are most efficient with large data sets, as there is always an overhead with initial setup. These frameworks coupled with MapReduce based designs provide a means to partition the analysis tasks effectively.

Hydra was designed to specifically take full advantage of the MapReduce architecture and native capabilities. The MapReduce algorithm parallelizes the performance of a number of problems. Computation is performed in two phases. The first phase, called the mapper, receives data in parallel at a series of nodes, and then emits data as a series of key-value pairs. The unit of data received and emitted is application dependent. In our case the first mapper receives information representing a single spectrum and emits the same.

**Figure 1:**
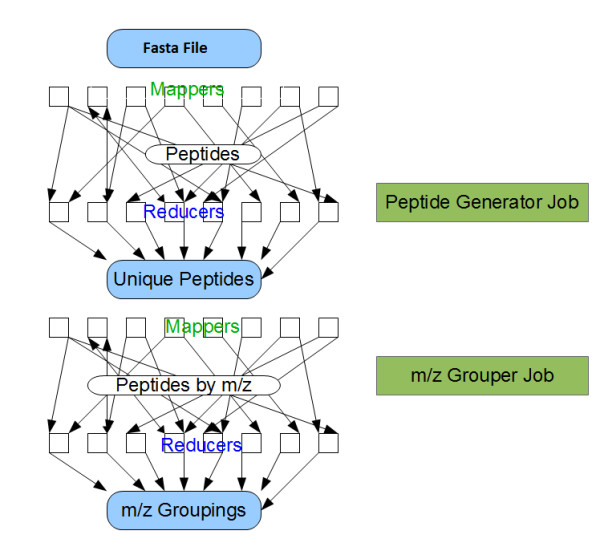
**MapReduce jobs to generate a list of peptides to score at a specified m/z ratio.** The first mapper generates all possible sequences and modified sequences defined in the search parameters for a given fasta database. The reducer eliminates duplicates, remembers all source proteins and emits the peptide with m/z as the key. The next set of reducers collects all peptides to be scored against a given m/z and stores them in the database.

**Figure 2:**
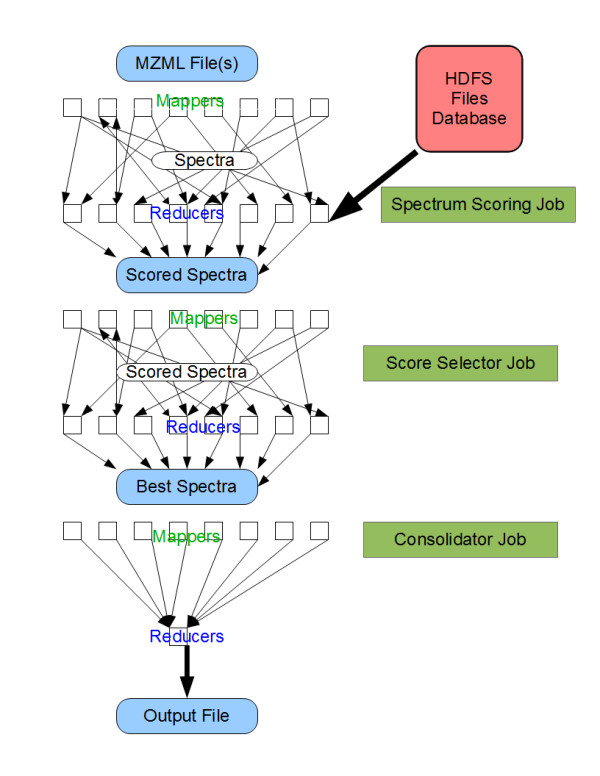
**MapReduce jobs to score measured spectra. Spectra are scored against the contents of the peptide database with a series of m/z values.** In the next job all scores are combined to generate the best scores. As a single file is the desired output, the last job has a single reducer allowing all output to go to a single file.

The reducers handle the key value pairs emitted by the mappers. The framework processes and sorts the data emitted by the mappers in order to offer the following guarantees: 1) all values associated with a specific key will be handled in a specific reduce task. 2) Any keys reaching a specific reducer will be received in sorted order. Each reducer may emit a collection of key-value pairs stored in a collection of files which is the same size as the number of reducers. The algorithms that are discussed in this paper take specific advantage of these features and are described in detail below. As with many other MapReduce based applications, our approach consists of a series of steps (see Figures 1 and 2) which are described in further detail below.

We use the Hadoop framework, which is the *de facto* open-source implementation of the MapReduce architecture supported by Apache and available on a number of machines. Our code is single threaded and uses the Hadoop framework to achieve parallelism in separate nodes on a cluster. One important feature of the approach is to precompute all candidate peptide sequences including any mass modifications and to group them by *m*/*z*. This allows each sequence and its corresponding spectrum to be generated only once.

Our implementation divides proteomic search into two tasks: generation of a peptide database and the subsequent scoring of a collection of spectra against the database. This separation allows the set of peptides with appropriate modifications to be generated in advance of searches and, as long as the set of proteins and modifications remains unchanged, to be reused in multiple searches. In addition, as discussed below, knowledge of the number of peptide ions to be scored allows tuning to control memory requirements.

### Generation of the peptide database

The peptide database is written to answer a single query: given a specific *m*/*z* ratio return a list of all peptides and modified peptides to score having that m/z. The task of generating peptides has two parts - the first finds a list of all unique peptides in a protein database after applying a specified digestive enzyme (e.g., trypsin allowing one missed cleavage) and a collection of modifications. The second element is to generate groups of peptides with a specified *m*/*z* ratio and store them.

Our MapReduce implementation (see Figure 1) has the following steps: for every protein, the mapper generates all peptides and uses the sequence (with modifications) as a key and an id of the protein as a value. Every key delivered to the reducer represents a unique sequence (duplicate keys are collapsed automatically) and the list of values represents the proteins containing the sequence. The reducer emits the sequence as the value with the *m*/*z* ratio as a key.

The next reducer receives all sequences scored at a specific *m*/*z* ratio and stores them for later analyses. Also generated is a list of *m*/*z* values and the corresponding number of scored sequences.

### Scoring spectra

The algorithm for scoring spectra uses three MapReduce jobs (Figure 2). The first job sends spectra to be scored against a set of peptide ions having a theoretical *m*/*z* ratio within the specified tolerance of the measured one. The second job combines all scoring of a specific spectrum, choosing the best scoring peptides. The third job combines the scoring of all spectra into a single output file.

These algorithms score spectra against a range of m/z values within a specific delta of the precursor *m*/*z* ratio. Our implementation assigns candidate peptides to a specific *m*/*z* group (1 Dalton for z = 1). Spectra are assigned to score against a set of groups close to the precursor ion *m*/*z*. Every group is scored on a separate reducer. When the number of peptides in a group exceeds a known limit, the group is split and scored on multiple machines. For each spectrum the top scoring peptides in the group, as well as scoring statistics are gathered as an object, serialized as XML and written with the scan id as the key.

The second MapReduce job gathers the scoring for each spectrum and collates the results. Peptide-spectrum matches (PSMs) from all groups are combined into a single list sorted by score. Again, the top scoring peptides and overall statistics are packed into a single object and serialized as XML with the scan id being the key.

The consolidation task receives these overall score objects for each scan. The data is reformatted into the standard X!Tandem output and written to a single file. Following the completion of the consolidation task, the output of the consolidator stage, a file in X!Tandem format is copied from the cluster to the user’s machine.

## Results and discussion

The program was tested against larger searches to validate scalability and the ability to handle large search spaces. We ran the program against three databases: a large non-redundant (nr) sample (~16.4 million proteins), a subset with half the original nr data and one with a quarter the original set. Sample data sets ranged from 4000 to 500,000 spectra. We used a cluster with 4 nodes with 8 cores/node, and each node had a terabyte disk. All nodes were in the same rack connected with standard Ethernet.

To generate the original database, we used NCBI's non-redundant list of protein sequences for building the database of peptides. This file contains all available protein sequences for all organisms that differ at least with one amino acid residue [17]. The smaller databases were generated by selecting subsets of the larger database. For searching against the human proteome, the 2012 February UniProt reference proteome (65835 proteins) was used [18].

The scalability problems with proteomics searching come down to three factors. The first factor is the size of the peptide database: the number and size of the proteins considered; whether tryptic only or semitryptic peptides are searched; and the number of modifications and missed cleavages. Secondly the size of the experiment, which is the number of spectra scored. The third factor is the number of computer resources that can be made available. Our algorithm is designed to be optimized to scale for all the three factors. So that there are no limits on the number of searched peptides, the number of scored spectra, and the number of compute nodes that can be used. Adding proteins or spectra will increase execution time for a given set of resources, but it does not prevent the algorithm from arriving at a solution.

**Figure 3:**
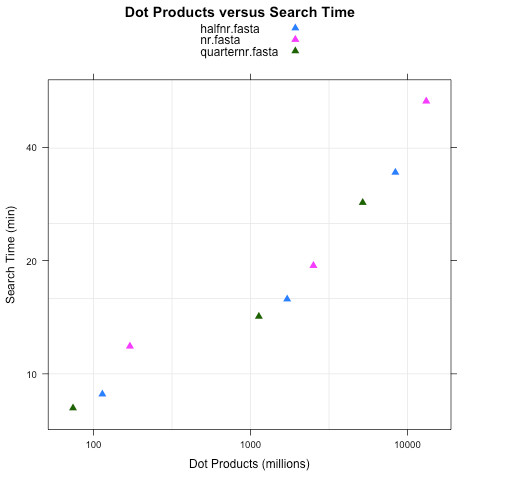
**Search times as a function of job complexity.** Complexity is measured as dot products - the score of one spectrum against one peptide. Complexity depends on the number of spectra, the size of the protein database and the modifications and cleavages searched. The measured spectra files used for benchmarking our implementation were picked out of the public experiments of the PRIDE (Proteomics Identifications Database) proteomics repository. The PRIDE accession numbers of the 3 experiments used for making Figure 3 are: 7962, 15459, 10295. The PRIDE xml files containing spectra were downloaded from the PRIDE website and were opened in the PRIDE Inspector [19]. The mgf export functionality of PRIDE Inspector was used to generate the mgf files used in the searches, with only human tissue samples or cell lines being used to generate the mass spectra.

**Figure 4:**
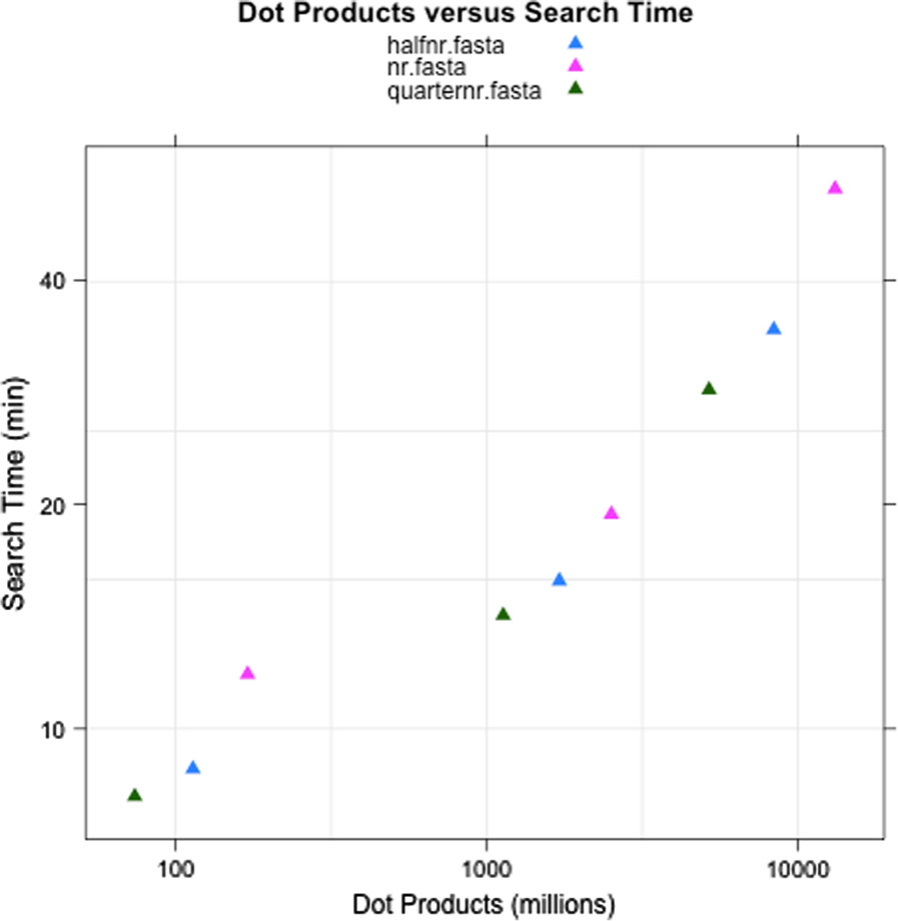
**Showing database build time as a function of the number of peptides cataloged.** The figure shows the time for building a tryptic database against the number of peptides. The data is for a tryptic database with limited modifications. Build times are higher with semitryptic builds or with more modifications. Build times for tryptic digests range from a few minutes, largely representing set up time, to under an hour for the largest databases with over a million proteins.

The performance of Hydra scales with the product of the number of spectra and the number of peptides considered (see Figure 3). Every time a measured spectrum is scored against a peptide, a dot product is computed. The number of dot products is a reasonable way to measure the complexity of a search, and depends on the number of spectra scored and the number of scored peptides close enough to the precursor *m*/*z*. Hydra performed 27 billion peptide scorings in approximately 40 minutes. For benchmarking we used: a dedicated 4 core linux server, for X!Tandem; and a 43 node Hadoop/Linux cluster with 8 cores per node (433 cores in total), was used for Hydra. The longest task (scoring nr) took approximately 50 minutes on the cluster, while it took almost 6 days using X!Tandem on a 4 core local machine. A smaller task (quarternr) which took 15 minutes on the cluster took 38 hours on a single four core machine (see Table 1). Because the peptide database is reusable in multiple searches we build the database in a separate job (see Figure 4).

**Table 1:** Comparison of search times for standard X!Tandem and Hydra

**Mode**	**Scans**	**Nodes (Cores)**	**DB Name**	**Proteins (K)**	**Peptides (M)**	**Dot product (M)**	**Tim (min)**
Hadoop	16000	43 (344)	ecoli	5.4	1.3	164	9.8
Hadoop	256000	43 (344)	ecoli	5.4	1.3	23395	338
Tandem	4663	1 (4)	human	222	168	477	29
Hadoop	4663	43 (344)	human	222	168	477	4.7
Tandem	184880	1 (4)	nr	4370	692	3291	2280
Hadoop	184880	43 (344)	nr	4370	692	3291	15.4
Tandem	184880	1 (4)	nr	16392	1248	13167	8410
Hadoop	184880	43 (344)	nr	16392	1248	13167	52.7

Example of comparison of run time for different complexities of search using the standard X!Tandem implementation and Hydra. The scans columns gives the number of spectra searched against, the Nodes column is the number of resources used (the first number of the number of machines, the second number is the number of total cores), the database name is the species database used, the Database Proteins is the number of proteins in the database, the dot product is the number of actual calculations. The times show that Hydra, unlike X!Tandem, is able to scale nearly linearly with the size of the problem. However, due to the startup costs associated with Hydra it is not suited for small searches. The PRIDE accession numbers for the spectra used were 10295 and 7962.

Hydra and X!Tandem do differ in the data that they generate, as X!Tandem returns a single peptide, and Hydra returns a list of the top likely peptides. When comparing the outputs of X!Tandem to Hydra with artificial spectra they are greater than 99.9% identical in terms of the best peptide found. When running the system on experimental data there are differences due to algorithmic details (e.g. integer rounding), which means that they are 75% identical in terms of the top peptide and score matching, and 95% of the time they both return peptides with similar scores. It is interesting to note that X!Tandem itself does not always generate exactly the same results, possible due to race conditions in the code base. Because we can afford to do a slightly wider search, Hydra can find peptides the X!Tandem does not (in nearly all the cases where the systems do not match Hydra finds a peptide with a substantially higher score than X!Tandem).

## Conclusion

Scalability of search operations in mass spectrometry is a critical concern. The size of proteome databases continues to grow especially as more modifications, post-translational changes, isotopic labeling and semi-and non-tryptic peptides are searched. Isotopic labeling and modifications geometrically expand the search space of peptide masses. The wider availability of faster mass spectrometers and the common practice of scoring data with multiple search algorithms are having huge effects on the computational requirements for such operations. The software discussed in this paper is designed to have few limits on scalability.

Search is inherently a parallel operation and algorithms already exist that have been adapted to work in parallel. In this paper we present the use of a distributed framework to develop a new generation of search algorithms, as these frameworks lend themselves conveniently to sequence-spectra matching. The advantage of using such a framework is that much of the infrastructure of managing parallel jobs is built into the framework. Hadoop has been demonstrated to scale up to thousands of processors. The details of handling a cluster, distributing work, gathering results and, critically for large systems, handling failure and retry are built into the framework.

The genomics field has already demonstrated the power of using cloud or high distributed frameworks for computational intensive tasks [20]. As the computational burden in tandem mass spectrometry proteomics based searching is large and increasing, exploration into the use of such distributed frameworks is necessary. This increase in search space is going to continue to be a rate limiting step, and this paper discusses one strategy which can be used to overcome these limitations.

## Availability

The full source code, documentation and test code is available at http://code.google.com/p/hydra-proteomics/.

## Competing interests

The authors declare that they have no competing interests.

## Authors’ contributions

JB, SL and SK conceived of the project. SL designed and implemented the work. ED, MH RM and AC helped in the design, and provided expert input. HH, SL and AC helped with testing and results generation. All authors read and approved the final manuscript.
